# Clinicopathological characteristics and diagnostic performance of metagenomic pathogen detection technology in mycobacterial infections among HIV patients

**DOI:** 10.3389/fcimb.2025.1584189

**Published:** 2025-04-29

**Authors:** Man Li, Jiamin Chen, Liang Zhang, Xiangmei Chen, Jianfeng Zhou, Feifei Liu, Xingang Zhou, Jiang Xiao, Kun Yang, Liming Qi, Xiaoyi Han, Ting Liu, Hongxin Zhao, Zhen Zhou, Xiaoyou Chen, Lei Sun

**Affiliations:** ^1^ Department of Pathology, Beijing Ditan Hospital, Capital Medical University, Beijing, China; ^2^ Department of Diagnostics, Beijing KingMed Center, Beijing, China; ^3^ Department of Clinical Laboratory, Guangzhou Kingmed Center, Guangzhou, China; ^4^ Center for Infectious Diseases, Beijing Ditan Hospital, Capital Medical University, Beijing, China; ^5^ Department of Interventional Catheterization, Beijing Ditan Hospital, Capital Medical University, Beijing, China

**Keywords:** mycobacterium tuberculosis, non-tuberculous mycobacteria, HIV, granulomas, necrosis

## Abstract

**Background:**

Mycobacterial infections represent a major cause of morbidity and mortality in HIV-infected individuals. This study evaluated diagnostic techniques for mycobacterial identification and compared clinicopathological features between HIV-positive and HIV-negative patients.

**Methods:**

We analyzed 88 tissue samples (with 41 matched blood and 28 sputum samples) using histopathology (HE and acid-fast staining), bacterial culture, MTB-PCR (sputum/biopsy), PCR-reverse dot blot hybridization (RDBH), and metagenomic pathogen detection technology (MetaPath™). Logistic regression analyses were performed to identify factors affecting detection rates.

**Results:**

Mycobacterial infection was detected in 95.5% (84/88) of patients. Among HIV-positive patients (n=63), 46% (29/63) had *Mycobacterium tuberculosis* (MTB) infections, and 44% (28/63) had non-tuberculous mycobacteria (NTM) infections, significantly higher than the 20% (5/25) NTM rate in HIV-negative patients. Univariate analysis identified HIV-positive status (*p*=0.009), lymph node involvement (*p*=0.020), and positive MetaPath™ results (*p*=0.002) as significant predictors of detection, while multivariate analysis confirmed these as independent factors (*p*=0.036; *p*=0.042; *p*=0.006). Lymph nodes were the most common infection site in HIV-positive patients (42.9%, 27/63), while lung tissue predominated in HIV-negative patients (48%, 12/25). MetaPath™ demonstrated superior sensitivity and specificity for detecting both MTB and NTM. Biopsy samples provided higher diagnostic accuracy than sputum or blood for lung and lymph node infections, but not for brain. In HIV-positive patients, NTM infections showed significantly more granuloma formation (*p*=0.032) and foam cells (*p*=0.005), but less necrosis (*p*=0.0005) compared to MTB infections. No significant differences were observed in HIV-negative patients.

**Conclusions:**

MetaPath™ is a highly effective diagnostic tool for mycobacterial infections, particularly in tissue biopsies. HIV-positive status, lymph node involvement, and MetaPath™ positivity independently predict mycobacterial detection. HIV-positive patients exhibit distinct clinicopathological features, emphasizing the need for tailored diagnostic and therapeutic approaches based on immune status.

## 1 Introduction

Mycobacterial diseases - tuberculosis (TB), non-tuberculous mycobacterial (NTM) infections, and leprosy - remain significant global health threats. TB continues to be the most impactful, with WHO reporting 10 million new cases and 1.5 million deaths annually (Hoffmann et al., 2023). Clinical manifestations range from classic pulmonary symptoms (chronic cough, fever, night sweats, weight loss) (Harding, 2020), to extrapulmonary disease (15-20% of cases) affecting multiple organ systems (Ketata et al., 2015). Extrapulmonary TB diagnosis remains challenging due to typically low bacterial loads in clinical specimens. NTM infections, particularly those caused by *Mycobacterium avium complex* and *M. abscessus*, have emerged as growing clinical concerns (Haworth et al., 2017). While primarily causing pulmonary disease, 20-30% of cases present as extrapulmonary infections, especially in immunocompromised hosts (Jamal and Hammer, 2022). Diagnostic interpretation is complicated by the frequent colonization of respiratory tracts in patients with chronic lung disease (Griffith et al., 2007). Leprosy, caused by *M*. leprae, maintains its distinctive clinical profile primarily involving the skin and peripheral nerves, though its global prevalence has significantly declined in recent decades (Maymone et al., 2020). The distinct pathological features and epidemiological patterns of these three mycobacterial diseases underscore the need for tailored diagnostic and therapeutic approaches.

The clinical management of mycobacterial diseases hinges on distinguishing latent infection from active disease, a framework best established for TB. Latent TB infection (LTBI) is defined by immunological evidence of Mycobacterium tuberculosis sensitization (positive TST or IGRA) without clinical or radiographic signs of disease (Getahun et al., 2015). In contrast, active TB requires both microbiological confirmation and compatible clinical manifestations (Lewinsohn et al., 2017). This distinction critically informs treatment decisions. For NTM, the concept of latency remains less clear. Positive cultures may indicate true infection or mere colonization, particularly in respiratory specimens from patients with chronic lung disease (Falkinham, 2011). This diagnostic ambiguity is especially challenging in immunocompromised patients, where treatment decisions must carefully integrate clinical symptoms, radiographic findings, and microbiological data.

Recent breakthroughs in molecular diagnostics have transformed mycobacterial disease detection. Nucleic acid amplification tests (NAATs), particularly the Xpert MTB/RIF and Xpert Ultra systems, now enable rapid (<2 hours), automated detection of Mycobacterium tuberculosis and rifampicin resistance (Dorman et al., 2018). These cartridge-based platforms integrate sample processing, DNA extraction, amplification, and real-time PCR detection, offering significant advantages over traditional smear microscopy and culture methods. The World Health Organization endorses these assays as first-line diagnostics for pulmonary and extrapulmonary TB across all age groups. Clinical studies demonstrate excellent sensitivity (89-98%) for smear-positive pulmonary TB and reliable performance (60-75%) in smear-negative cases (Acharya et al., 2020). Importantly, they maintain 65-80% sensitivity in HIV-positive patients with advanced immunosuppression, addressing a crucial diagnostic need in this high-risk population.

Despite technological advances, diagnosing and managing mycobacterial infections remains challenging, especially in immunocompromised hosts. HIV-infected individuals, a high-risk group, exhibit distinct immunological and pathological responses, often leading to atypical presentations, disseminated disease, and diagnostic difficulties (Geldmacher et al., 2008; Lawn et al., 2002; Barr et al., 2022). This study addresses these gaps by systematically evaluating contemporary diagnostic methods for active mycobacterial disease in HIV patients. By comparing multiple modalities, we aim to optimize detection strategies for pulmonary and extrapulmonary manifestations and refine clinical management protocols. Our findings will contribute to global efforts in combating these infections, particularly for high-burden immunocompromised populations.

## 2 Methods

### 2.1 Patients and samples

This prospective study analyzed 88 tissue specimens from patients with confirmed active mycobacterial disease at Beijing Ditan Hospital between April 2023 and December 2024. The cohort included 41 matched blood samples and 28 sputum specimens for comparative analysis. The inclusion criteria required active disease, defined by the presence of: (1) clinical symptoms (persistent cough ≥3 weeks, fever, night sweats, or >10% weight loss); (2) histopathological evidence of caseating granulomatous inflammation and/or characteristic radiographic findings; and (3) microbiological confirmation through positive culture, NAATs, or acid-fast staining. Exclusion criteria included latent infection, incomplete documentation, or confirmed alternative diagnoses. All specimens were processed within 2 hours using standardized protocols with uniform equipment and reagents. The study was approved by the Ethics Committee of Beijing Ditan Hospital (DTEC-KY2024-031-01), with patient data anonymized for confidentiality.

### 2.2 HE and acid-fast staining

Standard histopathological evaluation was performed on 4-μm formalin-fixed, paraffin-embedded (FFPE) tissue sections. Sections were stained with hematoxylin and eosin (H&E) using automated stainers (Leica ST5020) to assess tissue morphology and inflammatory patterns. For mycobacterial detection, acid-fast staining was conducted using a commercial kit (Zhuhai Beisuo Biotechnology, BA-4090B) following manufacturer’s protocol with modifications: tissue sections were stained with carbol fuchsin at 37°C for 18 minutes, decolorized in 1% acid-alcohol for 3 minutes, and counterstained with 0.3% methylene blue for 2 minutes. All stained slides were independently evaluated by two board-certified pathologists using standard light microscopy (Nikon Eclipse E200). A positive result required identification of ≥3 acid-fast bacilli per 100 high-power fields (400× magnification), appearing as 2-4 μm red, beaded rods against a blue background.

### 2.3 Histopathological evaluation

Histological features were systematically evaluated using a validated semi-quantitative scoring system. Granuloma formation was graded as follows: 0 (none), 1 (<3 granulomas per 100x field), 2 (3–10 granulomas per 100x field), and 3 (>10 granulomas per 100x field) (Cooper, 2009). Necrosis was categorized as 0 (none), 1 (<10%), 2 (10–50%), and 3 (>50% tissue involvement) (Russell et al., 2009). Inflammatory infiltration was similarly scored based on percentage of affected tissue area (Ehlers and Schaible, 2012). All sections were independently assessed by two experienced pathologists.

### 2.4 Real-time PCR detection of MTB

DNA was extracted from all 88 FFPE tissue samples and matched sputum specimens using commercial kits (AmoyDx Tissue Kit for FFPE samples; AmoyDx Cell Kit for sputum) following manufacturer’s protocols. The extraction process for FFPE tissues included deparaffinization with xylene, rehydration through graded alcohols, proteinase K digestion, and column-based purification. For sputum samples, DNA was isolated through chemical/enzymatic lysis followed by silica-membrane purification. DNA quality and quantity were assessed using NanoDrop spectrophotometry (A260/280 ratio 1.8-2.0 considered acceptable) and agarose gel electrophoresis. Real-time PCR was performed using a commercially available MTB detection kit (No. Z-RD-0060-02, Shanghai ZJ, China) targeting the IS6110 insertion sequence. Each 40μL reaction contained: PCR buffer, forward primer (5′-TCGCCCGTCTACTTGGTGTT-3′), reverse primer (5′-TGATGTGGTCGTAGTAGGTC-3′), Taq/UNG enzyme mix and 4 μL template DNA.

### 2.5 RDBH assay

The RDBH assay was conducted following the manufacturer’s protocol (NO. M202410001, Yaneng Bio, China). This method is a highly specific and robust molecular technique designed for the precise identification and differentiation of *Mycobacterium* species. The assay involves PCR amplification of conserved genomic regions, such as the 16S rRNA or *rpoB* genes, using universal primers, followed by hybridization of the amplified products to a membrane-immobilized probe array. These probes are tailored to recognize unique sequence variations among various *Mycobacterium* species, enabling simultaneous detection and discrimination based on distinct hybridization profiles. Owing to its high sensitivity, specificity, and rapid turnaround time, the RDBH assay has been widely adopted in clinical diagnostics for the accurate detection of MTB and NTM directly from clinical specimens. In this study, the assay incorporated a comprehensive panel of species-specific probes, covering a broad spectrum of mycobacterial species, including *Mycobacterium smegmatis* (MSM), *Mycobacterium intracellulare* (MIN), *Mycobacterium kansasii* (MKA), *Mycobacterium chelonae* (MCH), *Mycobacterium avium complex* (NMA), *Mycobacterium fortuitum* (MFO), *Mycobacterium terrae* (MTE), *Mycobacterium nonchromogenicum* (MNO), *Mycobacterium tuberculosis complex* (MTC), *Mycobacterium avium* (MAV), *Mycobacterium scrofulaceum* (MSC), *Mycobacterium abscessus* (MAB), *Mycobacterium xenopi* (MXE), *Mycobacterium gilvum* (MGI), *Mycobacterium phlei* (MPH), *Mycobacterium gordonae* (MGO), *Mycobacterium triviale* (MTR), *Mycobacterium gastri* (MGA), *Mycobacterium ulcerans* (MUA), *Mycobacterium szulgai* (MSZ), *Mycobacterium diernhoferi* (MDI), and *Mycobacterium simiae* (MSI).

### 2.6 MetaPath™ sequencing

DNA extraction from FFPE tissue sections (4 μm thick; 8 sections per sample) was performed as follows: Deparaffinization was carried out using a deparaffinization solution (56°C, 10 min, 400 rpm agitation), followed by centrifugation (15,000 rpm, 2 min). After supernatant removal, tissues were lysed in 750 μL ATL buffer supplemented with 20 μL proteinase K (56°C, overnight, 600 rpm orbital shaking). Lysates were homogenized with 250 μL of 0.5 mm glass beads (vortexing, 4000 rpm, 2 min), and DNA was purified using the MataPure™ DNA Extraction Kit (KS121-WSWTO-48) according to the manufacturer’s protocol. DNA libraries were prepared through mechanical fragmentation (target size: 350 bp), end-repair, adapter ligation, and PCR amplification. Eight uniquely barcoded libraries were pooled and enriched via the MetaPath Pathogen Capture Metagenomic Assay Kit (KingCreate, China), which employs biotinylated probes targeting 3,125 clinically relevant pathogens, including 1,850 bacterial, 692 fungal, 357 viral, and 226 parasitic species. Library quality was verified using an Agilent 2100 Bioanalyzer (peak size: 350 bp) and quantified via the Qubit dsDNA HS Assay Kit (Thermo Fisher). Sequencing was conducted on an Illumina MiniSeq platform (100-bp single-end reads, ≥300,000 reads/sample). For bioinformatic analysis, raw reads were demultiplexed (*bcl2fastq* v2.20.0.422), followed by adapter trimming and quality filtering (Fastp v0.23.1; Q-score ≥20, duplicate removal). Human-derived sequences were excluded by alignment to the GRCh38 reference genome (BWA v0.7.17-r1188). The remaining reads were mapped against a custom NCBI pathogen database (comprising 13,214 bacterial, 9,811 viral, 3,180 fungal, and 405 parasitic genomes) using an in-house pipeline. Pathogen identification was based on reads per million (RPM) normalization, with positivity thresholds set at RPM≥10 covering≥3 unique genomic regions. Alignments were visually confirmed using Integrated Genome Viewer (IGV).

### 2.7 Bacterial culture

A total of 28 freshly collected sputum specimens were processed for bacterial culture following standardized microbiological protocols. To minimize contamination while preserving mycobacterial viability, samples were decontaminated with 4% NaOH (15 min) and neutralized with phosphate-buffered saline (PBS, pH 7.4). After centrifugation (3,000 × g, 15 min), pellets were resuspended in PBS for subsequent culture inoculation. For comprehensive pathogen detection, each sample was cultured in parallel systems: (1) automated liquid culture using BacT/ALERT FA (aerobic) and FN (anaerobic) bottles (bioMérieux, France), with 0.5 mL aliquots monitored continuously in the BacT/ALERT 3D system; and (2) mycobacterial-specific culture with 0.3 mL aliquots inoculated into Mycobacteria Growth Indicator Tubes (MGIT; BD Diagnostics, USA). This dual-culture approach facilitated simultaneous detection of routine bacteria and acid-fast bacilli under optimized growth conditions.

### 2.8 Logistic regression assessed relationships between clinical factors and detection rates

Associations between clinical variables (HIV status, tissue type, and diagnostic method) and pathogen detection rates were evaluated using logistic regression analysis. Initial univariate analyses assessed each variable independently, with results reported as crude odds ratios (ORs) and 95% confidence intervals (CIs). Variables demonstrating potential associations (*p* < 0.05) in univariate analysis were subsequently included in multivariate modeling. The final multivariate model was constructed using backward stepwise selection with a retention threshold of *p* < 0.05, while adjusting for age and gender as potential confounders.

### 2.9 Statistical analysis

The statistical analysis was conducted with continuous variables expressed as mean ± standard deviation. Nonparametric continuous data were analyzed using the Mann-Whitney U test, while categorical variables were evaluated with either the chi-square test or Fisher’s exact test depending on the sample characteristics. Sample size determination was performed using the standard diagnostic test formula (n = Z² × p[1-p]/E²) with parameters set at 95% confidence level (Z = 1.96), expected sensitivity of 95% (p = 0.95), and 5% margin of error (E = 0.05), yielding a minimum requirement of 73 cases. After accounting for a 10% potential exclusion rate, the target sample size was increased to 80 cases. The study ultimately included 88 cases (63 HIV-positive and 25 HIV-negative), exceeding the calculated requirement. All statistical analyses were performed using SPSS software (version 21.0), with a significance threshold set at *p* < 0.05.

## 3 Results

### 3.1 Patient characteristics

The study population comprised 88 patients, with a predominance of HIV-positive cases (71.6%, 63/88) compared to HIV-negative controls (28.4%, 25/88). The overall cohort had a mean age of 44.1 ± 13.0 years (range: 19–71 years), with significant age differences observed between groups - HIV-positive patients were younger (41.5 ± 10.3 years) than their HIV-negative patients (50.0 ± 16.8 years; *p*=0.02). A striking gender disparity was noted, with males representing 93.7% (59/63) of the HIV-positive group versus 44% (11/25) in the HIV-negative group (*p*=0.001). As expected, HIV-positive patients demonstrated significantly elevated viral loads and reduced CD4+ T-cell counts compared to controls. Interestingly, histopathological examination revealed no significant intergroup differences in granuloma formation (*p*=0.999) or necrosis (*p*=0.999), suggesting comparable pathological manifestations regardless of HIV status (**Table 1**).

**Table 1:** Demographic and clinical characteristics of the HIV-positive and HIV-negative patients.

	HIV-positive	HIV-negative	*P* value ^a^
	(N=63)	(N=25)	
Age (years)	41.5 ± 10.3	50.0 ± 16.8	0.02^*^
Gender (n, %)			
Female	4 (6.7%)	14 (56.0%)	0.001^*^
Male	59 (93.7%)	11 (44.0%)	
Log2 (Viral Load+1)	19.1 ± 20.4	NA	NA
CD4 count (cells/μl)	41 ± 253.6	NA	NA
Granuloma (n, %)			
Yes	51 (80.9%)	21 (84.0%)	0.999
No	12 (19.1%)	4 (16.0%)	
Necrosis (n, %)			
Yes	38 (60.3%)	15 (60.0%)	0.999
No	25 (39.7%)	10 (40.0%)	

^a^HIV-positive vs HIV-negative; * *p*< 0.05. NA, Not Applicable.

### 3.2 Tissue tropism and pathogen distribution in HIV-positive and HIV-negative patients with mycobacterial infections

The study revealed distinct patterns of mycobacterial tissue tropism between HIV-positive and HIV-negative patients. Among HIV-positive individuals, lymph node represented the primary infection site (42.9%, 27/63), followed by lung (19.0%, 12/63), brain (11.1%, 7/63), and bronchus (11.1%, 7/63) involvement. In contrast, HIV-negative patients showed predominant lung localization (48.0%, 12/25), with significantly lower lymph node involvement (8.0%, 2/25). Comparative analysis demonstrated statistically significant differences in lung (*p*=0.009) and lymph node (*p*=0.002) tropism between groups, while brain (*p*=0.310) and GIT (*p*=0.4003) involvement showed comparable frequencies. Microbiological characterization identified mycobacterial infections in 95.5% (84/88) of cases. HIV-positive patients exhibited near-equivalent proportions of MTB (46.0%, 29/63) and NTM (44.4%, 28/63), with rare co-infections (3.2%, 2/63). The NTM spectrum in this group was dominated by MAV (20.6%, 13/63), MAB (4.8%, 3/63), MXE (1.6%, 1/63), MIN (4.8%, 3/63), MGO (1.6%, 1/63), MAV-MAB (6.3%, 4/63), MAB-MIN (3.2%, 2/63) and MAV-MKA (1.6%, 1/63). Conversely, HIV-negative patients showed MTB predominance (68.0%, 17/25), with lower NTM prevalence (20.0%, 5/25) and higher co-infection rates (12.0%, 3/25). Statistical analysis confirmed significantly greater NTM frequency in HIV-positive patients (*p*=0.0497), while MTB (*p*=0.097) and co-infection (*p*=0.36) trends did not reach significance (**Table 2**).

**Table 2:** Comparative analysis of mycobacterial infections across tissues in HIV-positive versus HIV-negative patients.

	Total (N=88)	HIV-positive (N=63)	HIV-negative (N=25)	*P* value ^a^
Tissue types				
Lung	24 (27.3%)	12 (19.0%)	12 (48.0%)	0.009^*^
Lymph node	29 (33.0%)	27 (42.9%)	2 (8.0%)	0.002^*^
Brain	12 (13.6%)	7 (11.1%)	5 (20.0%)	0.3097
GIT	7 (8.0%)	4 (6.3%)	3 (12.0%)	0.4003
Bronchus	8 (9.1%)	7 (11.1%)	1 (4.0%)	NA
Vertebral canal	4 (4.6%)	3 (4.8%)	1 (4.0%)	NA
Adrenal gland	1 (1.1%)	0	1 (4.0%)	NA
Mediastinum	1 (1.1%)	1 (1.6%)	0	NA
Great Omentum	1 (1.1%)	1 (1.6%)	0	NA
Appendix	1 (1.1%)	1 (1.6%)	0	NA
Mycobacterial infections				
MTB	46 (52.3%)	29 (46.0%)	17 (68.0%)	0.097
NTM	33 (37.5%)	28 (44.4%)	5 (20.0%)	0.0497*
MAV	14 (16.0%)	13 (20.6%)	0	
MAB	4 (4.5%)	3 (4.8%)	1 (4.0%)	
MXE	2 (2.3%)	1 (1.6%)	1 (4.0%)	
MIN	3 (3.4%)	3 (4.8%)	0	
MFO	1 (1.1%)	0	1 (4.0%)	
MGO	1 (1.1%)	1 (1.6%)	1 (4.0%)	
MAV-MAB	5 (5.7%)	4 (6.3%)	1 (4.0%)	
MAB-MIN	2 (2.3%)	2 (3.2%)	0	
MAV-MKA	1 (1.1%)	1 (1.6%)	0	
Co-infection (MTC-NTM)	5 (5.7%)	2 (3.2%)	3 (12%)	0.36
MTC-MAB	4 (4.5%)	2 (3.2%)	2 (12.0%)	
MTC-MXE	1 (1.1%)	0	1 (4.0%)	
Non- infections	4 (4.5%)	4 (6.4%)	0	NA

^a^HIV-positive vs HIV-negative; * *p*< 0.05. NA, Not Applicable.

### 3.3 Analysis of the diagnostic accuracy across methods

**Figure 1:**
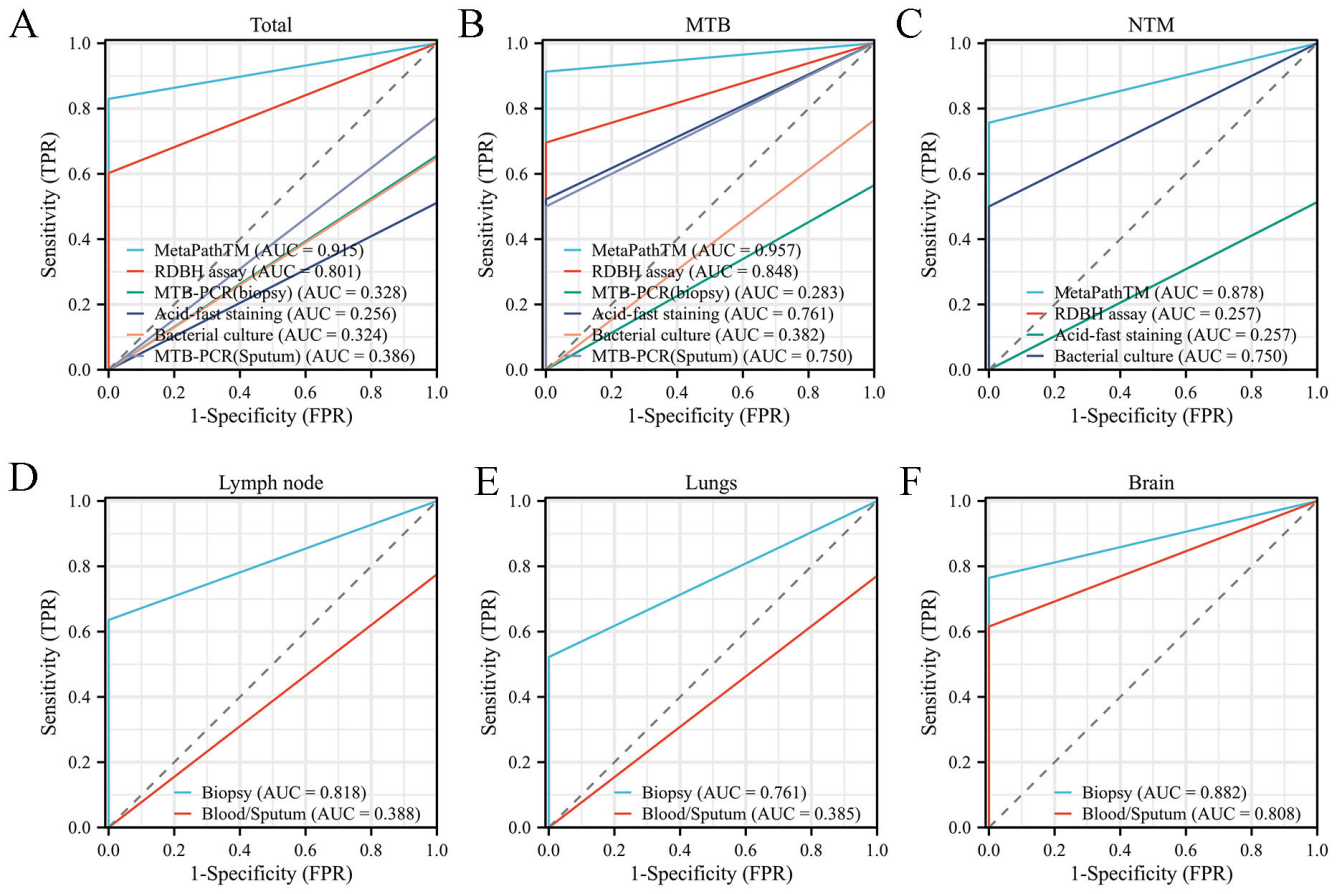
Comparative diagnostic performance analysis using ROC curve methodology. **(A-C)** ROC curve evaluation of diagnostic efficacy for: **(A)** total mycobacterial infections **(B)** MTB **(C)** NTM. **(D-F)** Comparative ROC analysis of diagnostic accuracy between tissue biopsy specimens and sputum/blood samples in:**(D)** lymph node **(E)** lungs **(F)** brain manifestations.

The diagnostic performance of various detection methods was evaluated against pathological diagnosis and clinical treatment outcomes as the reference standard. MetaPath™ demonstrated superior sensitivity (92.8%) and perfect specificity (100%), significantly outperforming conventional methods including bacterial culture (35.7% sensitivity, 62.5% specificity), MTB-PCR (30.3-34.0% sensitivity, 60-100% specificity), acid-fast staining (52.7% sensitivity, 64.3% specificity), and RDBH assay (66.2% sensitivity, 71.4% specificity). Statistical comparison revealed significant differences in sensitivity (χ²=100.7, *p*<0.001) but not specificity (χ²=12.24, *p*=0.128) across methods (**Table 3**). MetaPath™ showed excellent discriminatory power with AUC values of 0.915 for overall infection detection, 0.957 for MTB-specific diagnosis, and 0.878 for NTM identification (**Figures 1A–C**). Comparative analysis of sample types demonstrated the diagnostic superiority of tissue biopsies over blood/sputum specimens, particularly for lymph node (AUC 0.818 vs 0.388, *p*<0.001) (**Figure 1D**) and lung (AUC 0.761 vs 0.385, *p*<0.001) infections (**Figure 1E**). While brain tissue biopsies showed higher AUC values (0.882 vs 0.808), this difference did not reach statistical significance (*p*=0.356) (**Figure 1F**). These results demonstrate that tissue biopsies from lungs and lymph nodes are essential for optimal mycobacterial diagnosis, as they yield significantly higher detection rates than blood or sputum samples. In contrast, for suspected brain infections, both biopsy and blood testing may be diagnostically comparable, allowing flexibility based on clinical feasibility and suspected pathogen.

**Table 3:** Diagnostic accuracy of various methods for *Mycobacterium* Detection.

	Sensitivity (95% CIs)	Specificity (95% CIs)	PPV (95% CIs)	NPV (95% CIs)	Accuracy (95% CIs)
Bacterial culture%	35.7 (19.2–56.4)	62.5 (25.9–89.8)	76.9 (49.7–91.8)	41.7 (26.7–58.4)	41.7 (26.7–58.4)
MTB-PCR% (Sputum)	30.3 (16.6–48.6)	100 (72.2–100)	100 (72.2–100)	30.3 (16.6–48.6)	60.6 (43.9–75.3)
Acid-fast staining%	52.7 (41.1–64.0)	64.3 (36.6–84.7)	88.6 (76.0–95.1)	20.1 (10.6–34.5)	54.5 (44.3–64.4)
MTB-PCR% (biopsy)	34.0 (22.1–48.4)	60 (27.4–86.3)	80.9 (59.9–92.4)	15.4 (6.9–30.4)	38.3 (27.0–51.2)
RDBH assay%	66.2 (54.6–76.1)	71.4 (42.0–90.4)	92.4 (82.1–97.0)	28.6 (15.9–46.0)	67.0 (56.7–76.0)
MetaPath™%	92.8 (85.2–96.7)	100 (51.7–100)	100 (95.4–100)	40 (14.7–72.4)	93.2 (85.8–97.0)

MTB, *Mycobacterium tuberculosis*; RDBH, PCR-reverse dot blot hybridization, PPV, positive predictive value, NPV, negative predictive value, PCR, polymerase chain reaction; CIs, confidence intervals.

### 3.4 Pathological characteristics of mycobacterial infections in patients with and without HIV

**Figure 2:**
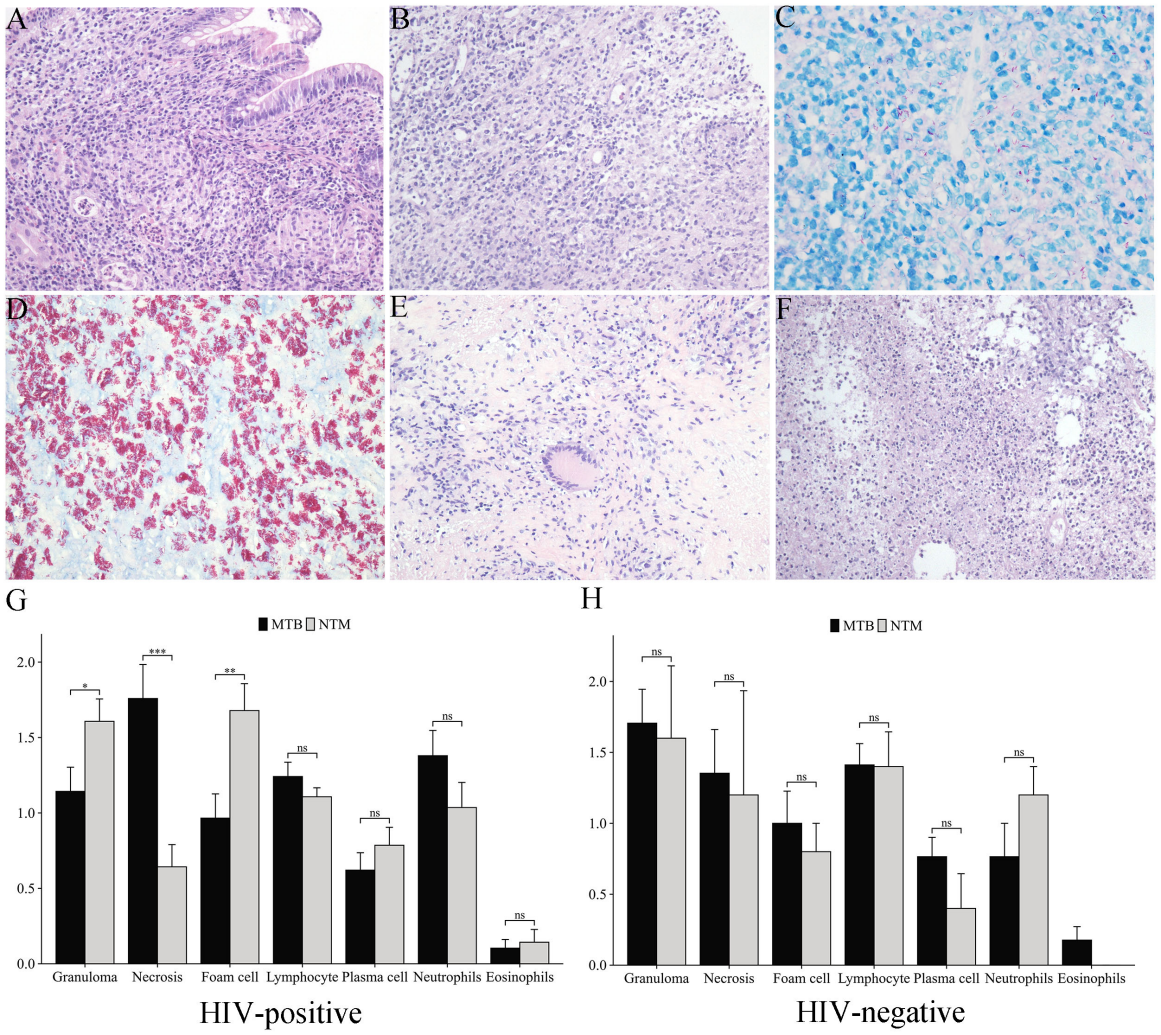
Histopathological features of mycobacterial infections. **(A)** Typical granulomas in intestinal mucosa stroma (HE staining, 200×). **(B)** Atypical granulomas with histiocyte proliferation and lymphocyte/plasma cell infiltration (HE staining, 200×). **(C)** MTB (acid-fast staining, 400×). **(D)** *Mycobacterium avium* (MAV) (acid-fast staining, 400×). **(E)** Caseous necrosis with multinucleated giant cells (HE staining, 200×). **(F)** Purulent necrosis with neutrophil infiltration (HE staining, 200×). **(G, H)** Comparative analysis of inflammatory cell infiltration patterns in MTB vs. NTM infections: **(G)** HIV-positive patients, **(H)** HIV-negative patients. *p<0.05, **p<0.01, ***p<0.001, ns, not significant (p≥0.05).

The primary pathological features of mycobacterial infections included granuloma formation, caseous necrosis, histocytosis, foam cells, multinucleated giant cells formation, and inflammatory cell infiltration such as lymphocytes, plasma cells, neutrophils and eosinophils. Typical granulomas were characterized by epithelioid cells, multinucleated giant cells, and surrounding lymphocytes and fibroblasts (**Figure 2A**). In contrast, atypical granulomas exhibited nonspecific inflammatory changes, such as histiocyte, plasma cell, and lymphocyte infiltration (**Figure 2B**). Both MTB and NTM were acid-fast positive, appearing red under staining. MTB typically exhibited a slender, slightly curved, branching morphology and was distributed diffusely throughout tissues (**Figure 2C**), while NTM was predominantly localized within the cytoplasm of foam cells (**Figure 2D**). 60.2% of samples displayed varying degrees of necrosis, including caseous necrosis with multinucleated giant cells (**Figure 2E**) and purulent necrosis with neutrophil infiltration (**Figure 2F**). The histopathological analysis revealed distinct inflammatory patterns between MTB and NTM infections in HIV-positive patients. NTM infections were characterized by significantly more pronounced granulomatous inflammation (*p*=0.032) and foam cell formation (*p*=0.005) compared to MTB infections. In contrast, MTB infections demonstrated markedly higher frequencies of necrosis (*p*=0.0005). However, no significant differences were observed in other inflammatory cell infiltrates (lymphocytes, plasma cells, neutrophils, or eosinophils) between the two infection types (**Figure 2G**). Notably, HIV-negative patients showed no significant histopathological differences between MTB and NTM infections in terms of granuloma formation, necrosis, or inflammatory cell profiles (**Figure 2H**).

### 3.5 Differential inflammatory infiltration in mycobacterial infections

**Figure 3:**
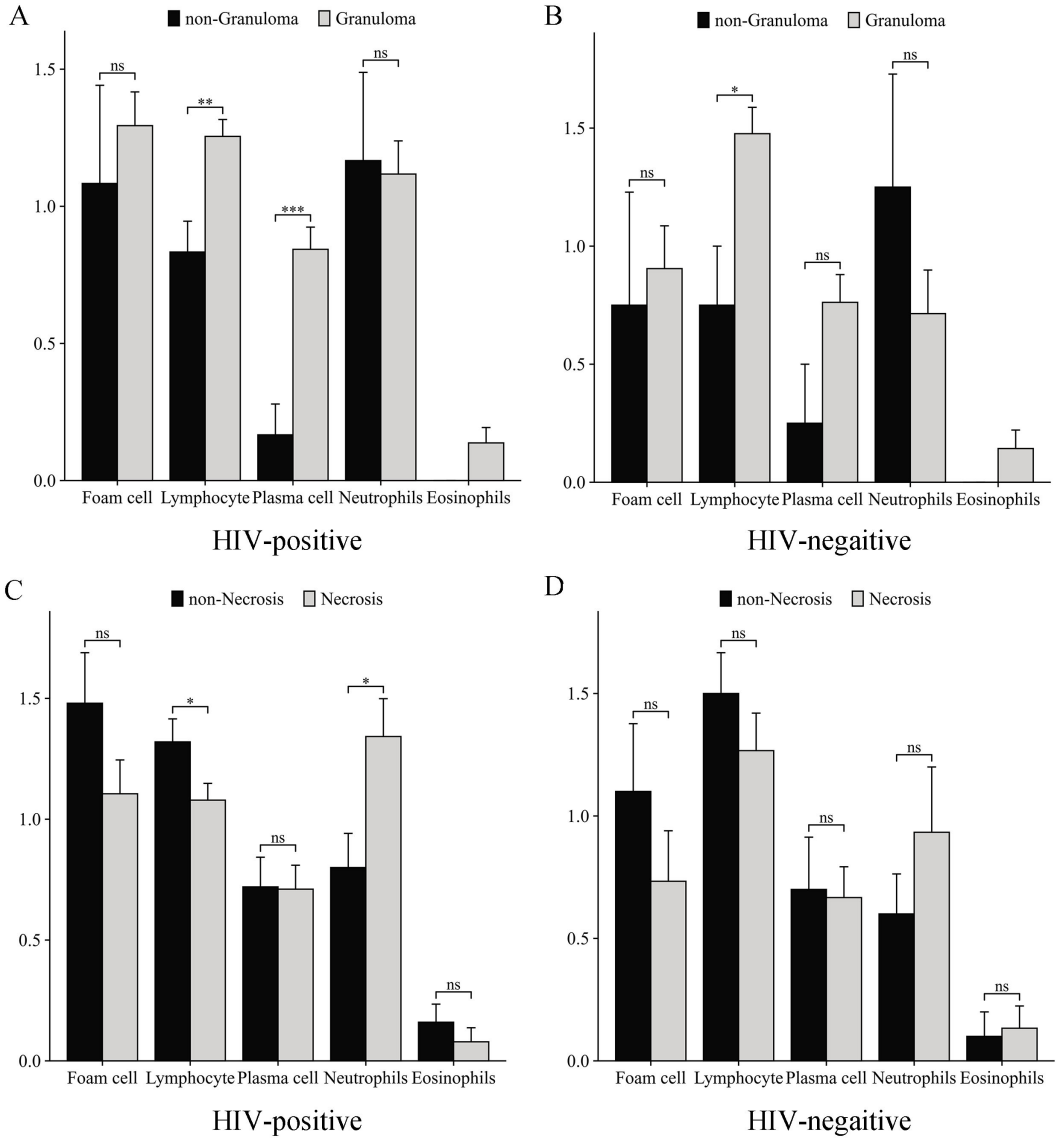
Inflammatory cell infiltration patterns in mycobacterial infections. Comparative analysis of inflammatory cell infiltration in granulomatous vs. non-granulomatous tissues: **(A)** HIV-positive patients, **(B)** HIV-negative patients. Inflammatory profiles in necrotic vs. non-necrotic tissues: **(C)** HIV-positive patients, **(D)** HIV-negative patients. *p<0.05, **p<0.01, ***p<0.001, ns, not significant (p≥0.05).

Comparative analysis of inflammatory cell infiltration in granulomatous and non-granulomatous tissues revealed that lymphocytes constituted the predominant inflammatory cell population in both HIV-positive (**Figure 3A**) and HIV-negative (**Figure 3B**) patients during granuloma formation. Notably, plasma cells were found to contribute to granuloma development in HIV-positive individuals. Further examination of necrotic versus non-necrotic regions demonstrated that neutrophils were significantly more abundant in necrotic areas of HIV-positive patients (*p*=0.030), whereas lymphocyte infiltration was markedly reduced (*p*= 0.040) (**Figure 3C**). In contrast, HIV-negative patients exhibited no significant differences in the distribution of foam cells, lymphocytes, plasma cells, neutrophils, or eosinophils between necrotic and non-necrotic regions (**Figure 3D**).

### 3.6 Logistic regression analysis of factors affecting mycobacterial detection rates

**Figure 4:**
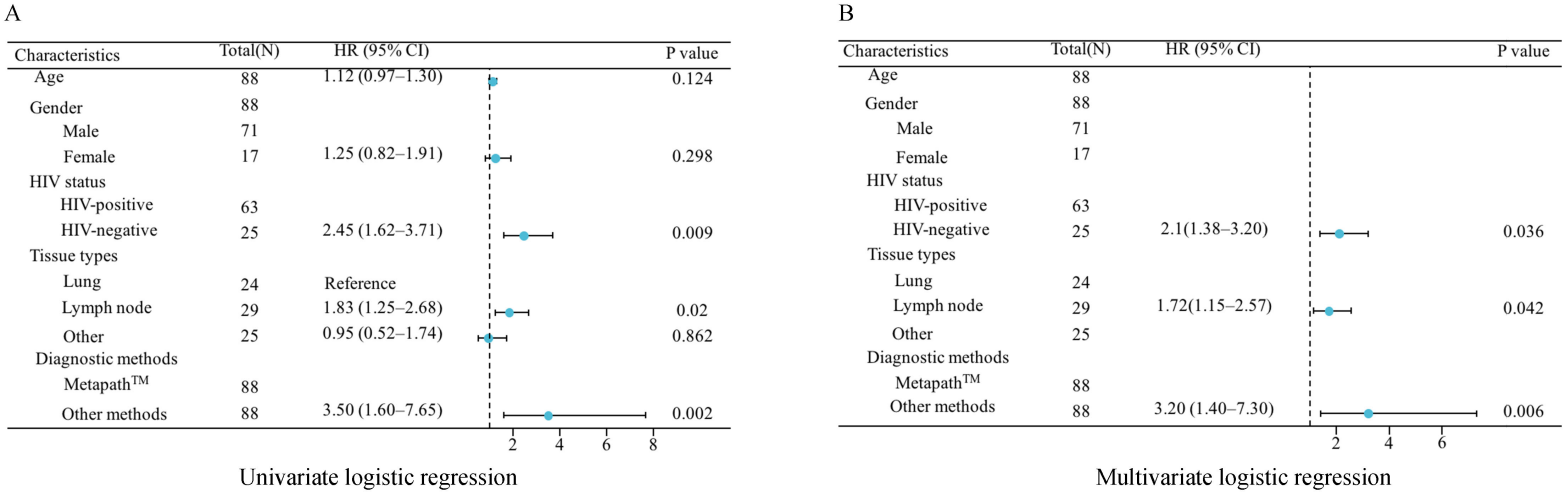
Logistic regression analysis of factors influencing mycobacterial detection rates. **(A)** Univariate logistic regression analysis **(B)** Multivariate logistic regression analysis.

Both univariate and multivariate logistic regression analyses were conducted to identify factors influencing mycobacterial detection rates. The univariate analysis revealed several significant associations: HIV-positive status (*p*=0.009), lymph node involvement (*p*=0.020), and positive MetaPath™ results (p=0.002) were all significantly correlated with higher detection rates (**Figure 4A**). Notably, neither age (*p*=0.124) nor gender (*p*=0.298) demonstrated significant associations. In the multivariate analysis adjusting for potential confounders, HIV-positive status (*p*=0.036), lymph node involvement (*p*=0.042), and positive MetaPath™ results (*p*=0.006) emerged as independent predictors of mycobacterial detection (**Figure 4B**).

## 4 Discussion

Consistent with previous reports, our study demonstrates distinct mycobacterial infection patterns between HIV-positive and HIV-negative individuals. While HIV-negative patients predominantly present with MTB infections, HIV-positive individuals exhibit comparable rates of MTB (46%) and NTM infections (44%), aligning with prior studies reporting NTM prevalence of 43-57.2% in this population (McCarthy et al., 2012; Singh et al., 2022). Notably, MAV accounted for half of all NTM cases among HIV patients - a clinically critical finding given its propensity to cause severe disseminated disease in immunocompromised hosts (Aliberti et al., 2024; Wetzstein et al., 2024). HIV patients with MAV infections have a mortality rate 1000 times higher than those without HIV (Fernandez-Diaz et al., 2025; Procop, 2017), underscoring the importance of considering MAV in the diagnosis and treatment of mycobacterial infections in HIV patients.

Our study provides a comprehensive evaluation of diagnostic modalities for mycobacterial infections, highlighting significant advancements and persistent challenges in clinical microbiology. While bacterial culture remains the gold standard, its clinical utility is limited by prolonged turnaround times (4–8 weeks) and suboptimal sensitivity (8.3% for MTB and 50% for NTM), consistent with previous reports (Batyrshina and Schwartz, 2019). Similarly, WHO-recommended immunodiagnostic tools (IGRAs and TB-Ab) demonstrate restricted clinical applicability due to their inability to differentiate NTM or diagnose active disease (Zhu et al., 2019). Tissue based diagnostics, including acid-fast staining, MTB-PCR, and RDBH, showed improved performance over blood or sputum testing, yet each presents notable limitations. Acid-fast staining, while widely accessible, suffers from poor sensitivity and cannot discriminate between MTB and NTM (Kong et al., 2021). MTB-PCR exhibits reagent-dependent sensitivity and fails to detect NTM species (Cho et al., 2015). Although RDBH offers superior accuracy for fresh specimens, its sensitivity declines with FFPE samples, likely due to nucleic acid degradation and contamination (Wu et al., 2009). The MetaPath™ platform demonstrated superior diagnostic performance with 92.8% sensitivity, significantly outperforming conventional methods (30.3-66.2%). This probe capture-based mNGS technology addresses critical limitations of traditional assays by requiring minimal DNA input (<5 ng), providing rapid results (24–48 hours), and enabling simultaneous detection of MTB, NTM and polymicrobial infections (Gu et al., 2019). While the platform shows excellent potential, particularly for challenging FFPE specimens, clinicians should consider its detection limits (<10 CFU/mL for bacteria) and implement strict contamination controls (Jacob et al., 2019; Mitchell and Simner, 2019). Optimal utilization requires integration with clinical and histopathological findings, especially in immunocompromised patients or atypical presentations. These advantages establish MetaPath™ as a valuable diagnostic tool for complex mycobacterial infections, warranting further validation in diverse clinical settings.

Lymph node involvement represents a predominant manifestation of extrapulmonary mycobacterial infections, observed in 80-90% of cases (Haridas et al., 2022). Our findings demonstrate distinct clinicopathological patterns between HIV-positive and HIV-negative patients, with significantly higher lymph node involvement in HIV-infected individuals (42.9% vs 8%). This HIV-associated tropism results from multiple pathophysiological mechanisms, including CD4+ T cell depletion-induced lymph node architectural disruption (Lawn et al., 2002), HIV-related fibrosis impairing lymphatic drainage, and gut barrier dysfunction facilitating bacterial dissemination (Brenchley and Douek, 2008; Chai et al., 2020). In contrast, immunocompetent hosts typically develop localized lungs infections due to preserved mucosal defenses. The diagnostic challenges are particularly evident in early-stage infections, where nonspecific clinical presentations complicate detection. Our results highlight the superior accuracy of tissue-based diagnosis compared to blood or sputum testing, underscoring the importance of biopsy in suspected cases. These findings support the need for HIV status-adapted diagnostic strategies, with particular emphasis on lymph node evaluation in immunocompromised patients. Future studies should investigate minimally invasive techniques to improve early detection while maintaining diagnostic reliability.

Patients with HIV infection exhibit significant differences in granuloma formation, necrosis patterns, and foam cell infiltration when comparing MTB and NTM infections, whereas these pathological distinctions are not observed in HIV-negative individuals. This differential presentation stems from distinct immune response pathways triggered by these mycobacterial pathogens. MTB infection induces a robust Th1-type immune response characterized by excessive production of proinflammatory cytokines including TNF-α, IFN-γ, and IL-1β (O’Garra et al., 2013). This hyperactive immune state leads to massive recruitment and activation of macrophages, neutrophils, and cytotoxic T cells at infection sites. The resulting oxidative stress and proteolytic enzyme release frequently cause caseous necrosis due to uncontrolled tissue damage (Collins et al., 2021). In contrast, NTM infection elicits a more modulated immune response, attributable to both lower pathogenicity and differences in cell wall components (Silwal et al., 2021). This milder immune activation promotes alternative macrophage differentiation, resulting in prominent foam cell accumulation - a hallmark of NTM pathology caused by impaired phagolysosomal fusion and dysregulated lipid metabolism (Peyron et al., 2008). These lipid-laden macrophages secrete an anti-inflammatory cytokine profile (IL-10, TGF-β, CCL2) that establishes a chemotactic gradient, recruiting diverse immune cells and fibroblasts to infection sites (Prasla et al., 2020). Fibroblast activation through PDGF and TGF-β signaling drives extracellular matrix deposition (collagen I/III, fibronectin, proteoglycans) (Nakajima et al., 2021; Wynn, 2008). Combined with controlled inflammation, this results in well-organized granulomas that effectively contain NTM pathogens while minimizing tissue destruction, explaining the characteristic histological features of NTM infection. Our findings also highlight lymphocytes as crucial mediators of granuloma formation in all patients, with plasma cells playing an additional compensatory role in HIV-positive individuals. These observations demonstrate preserved immune responses against mycobacterial infections regardless of HIV status, though further research is needed to elucidate the underlying molecular mechanisms and therapeutic implications.

Our study demonstrated the critical importance of lymph node evaluation in HIV-positive patients with suspected mycobacterial infection, demonstrating significantly higher detection rates in this anatomical site. The MetaPath™ assay emerges as a particularly effective diagnostic tool for high-risk populations, showing strong independent association with pathogen detection. These results suggest that integrating HIV status assessment with targeted lymph node examination could enhance early detection strategies, offering a practical approach to improve diagnostic efficiency in resource-limited settings. The preferential involvement of lymph nodes in HIV-associated mycobacterial infections likely reflects the altered immune surveillance and impaired granuloma formation characteristic of HIV-related immunosuppression. This anatomical predilection, combined with the superior sensitivity of molecular assays like MetaPath™, supports a targeted diagnostic approach that may overcome challenges posed by pauci-bacillary infections in this population. These findings have important clinical implications, particularly for settings where advanced diagnostic resources are scarce. By prioritizing lymph node sampling in HIV-positive individuals and utilizing sensitive molecular tests, clinicians may achieve more timely diagnoses while optimizing resource utilization. Future studies should evaluate the cost-effectiveness and implementation feasibility of this approach across diverse healthcare settings.

In summary, this study elucidates key clinicopathological features and diagnostic approaches for mycobacterial infections in HIV co-infected patients. While demonstrating the efficacy of current detection methods, our findings highlight the need for optimized management strategies. Study limitations include the short follow-up duration and moderate sample size, which warrant validation through larger multicenter studies with extended observation periods. These results provide a foundation for developing tailored diagnostic algorithms and underscore important directions for future research in this vulnerable population.

## Data Availability

The raw data supporting the conclusions of this article will be made available by the authors, without undue reservation.
